# Intraoperative radiotherapy with low-energy x-rays after neurosurgical resection of brain metastases—an Augsburg University Medical Center experience

**DOI:** 10.1007/s00066-021-01831-z

**Published:** 2021-08-20

**Authors:** Klaus-Henning Kahl, Nikolaos Balagiannis, Michael Höck, Sabine Schill, Zoha Roushan, Ehab Shiban, Heiko Müller, Ute Grossert, Ina Konietzko, Björn Sommer, Christoph J. Maurer, Ansgar Berlis, Volkmar Heidecke, Tilman Janzen, Georg Stüben

**Affiliations:** 1 Klinik für Strahlentherapie und Radioonkologie, Universitätsklinikum Augsburg, Stenglinstraße 2, 86156 Augsburg, Germany; 2 Medizinische Physik und Strahlenschutz, Universitätsklinikum Augsburg, Augsburg, Germany; 3 Klinik für Neurochirurgie, Universitätsklinikum Augsburg, Augsburg, Germany; 4 Klinik für Diagnostische und Interventionelle Radiologie und Neuroradiologie, Universitätsklinikum Augsburg, Augsburg, Germany

**Keywords:** IORT, Radionecrosis, Local control, Postoperative, Cavity boost

## Abstract

**Purpose:**

External-beam radiotherapy (EBRT) is the predominant method for localized brain radiotherapy (LBRT) after resection of brain metastases (BM). Intraoperative radiotherapy (IORT) with 50-kV x‑rays is an alternative way to focally irradiate the resection cavity after BM surgery, with the option of shortening the overall treatment time and limiting normal tissue irradiation.

**Methods:**

We retrospectively analyzed the outcomes of all patients who underwent neurosurgical resection of BM and 50-kV x‑ray IORT between 2013 and 2020 at Augsburg University Medical Center.

**Results:**

We identified 40 patients with 44 resected BM treated with 50-kV x‑ray IORT. Median diameter of the resected metastases was 2.8 cm (range 1.5–5.9 cm). Median applied dose was 20 Gy. All patients received standardized follow-up (FU) including 3‑monthly MRI of the brain. Mean FU was 14.4 months, with a median MRI FU for alive patients of 12.2 months. Median overall survival (OS) of all treated patients was 26.4 months (estimated 1‑year OS 61.6%). The observed local control (LC) rate of the resection cavity was 88.6% (estimated 1‑year LC 84.3%). Distant brain control (DC) was 47.5% (estimated 1‑year DC 33.5%). Only 25% of all patients needed WBI in the further course of disease. The observed radionecrosis rate was 2.5%.

**Conclusion:**

IORT with 50-kV x‑rays is a safe and appealing way to apply LBRT after neurosurgical resection of BM, with low toxicity and excellent LC. Close MRI FU is paramount to detect distant brain failure (DBF) early.

## Introduction

In recent decades, improved systemic treatment options have led to prolonged survival of patients suffering from metastatic cancer of many tumor entities (e.g., malignant melanoma, colorectal carcinoma, lung cancer), including patients with BM. In the early 1990s, the median survival of highly selected patients treated with resection of single BM and whole-brain radiotherapy (WBI) was 9.2 months [1]. In recent series of patients undergoing surgery and focal radiotherapy for BM, median survival even exceeds 24 months [2, 3]. As the majority of these patients live longer than 1 year after BM treatment, long-term LC and treatment-related long-term neurotoxicity have gained increasing importance. Several trials have shown the detrimental effect of WBI on neurocognitive functioning [4–8]. This led to a shift of paradigm in radiotherapy treatment after resection of BM, away from WBI and towards focal irradiation of the resection cavity [9, 10]. Most of this LBRT is administered via EBRT, either as stereotactic radiosurgery (SRS) or as hypofractionated stereotactic radiotherapy (HSRT) [11]. IORT with 50-kV x‑rays is an alternative method to irradiate the resection cavity focally after neurosurgical resection of BM [12]. Since 2013, we have treated patients with IORT after neurosurgical BM resection on an individual case-by-case treatment decision basis, always after discussion at and interdisciplinary consensus of the multidisciplinary tumor board (MTB). An expert panel of the German Society for Radiation Oncology (DEGRO) considered IORT after resection of BM as standard of care in the year 2017 [Expert panel decision DEGRO, inquiry 123, 17.02.2017]. Since then, the Augsburg University Medical Center (UKA) has run a program offering IORT to patients routinely scheduled for brain metastasectomy as an alternative to postoperative external-beam LBRT. A mean of 196 patients (SD 16 patients) were treated annually for BM at UKA in the past 3 years (2018–2020). Most of these patients (mean 88.5%, SD 1.4%) were treated with EBRT only, either with WBI or SRS/HSRT. A mean of 24 patients per year (SD 4 patients) were treated with a neurosurgical metastasectomy in this time period. Of those patients, approximately 38% were treated with IORT, all others with postoperative focal HSRT to the resection cavity.

## Materials and methods

We conducted a retrospective analysis of all patients who were treated with IORT after neurosurgical resection of BM between 2013 and 2020 at UKA. We identified all patients from our oncology information system MOSAIQ (ELEKTA AB, Stockholm, Sweden) and gained additional information via the hospital information system ORBIS (DEDALUS Healthcare Group AG, Bonn, Germany) and the radiology information and picture archiving and communication system DeepUnity (DEDALUS Healthcare Group AG). The timepoint for the last FU included in this analysis was February 5, 2021.

Treatment of all cases followed the recommendations of the UKA multidisciplinary tumor board (MTB). With regard to patient section, a minimal distance of 5 mm between the border of the contrast-enhancing lesion in MRI and the optic tract/brainstem was mandatory. Patients with a history of small-cell lung cancer were excluded. Depending on the decision of the neurosurgeon, some but not all patients with centrally located metastases or metastases in the posterior fossa were excluded. After informed consent of the patient, neurosurgical brain metastasectomy was performed, and a frozen section to confirm malignancy of the removed tumor was prepared. Hereafter, the resection cavity was irradiated with 50-kV x‑rays via an INTRABEAM system (ZEISS MEDITEC AG, Oberkochen, Germany) equipped with spherical applicators. The device and procedure have been described previously [12, 13]. Spherical applicator sizes of this IORT system range from 15 to 50 mm in diameter in 5‑mm increments. The suitable applicator size was chosen by the neurosurgeon and radio-oncologist according to the size of the resection cavity, providing direct contact of the cavity walls to the surface of the applicator. Radiation dose was prescribed to the surface of the applicator (tissue depth 0 mm), corresponding to the target volume/dose concept of postoperative SRS cavity treatment (GTV = CTV = cavity). Due to the dose distribution of the system, a 2-mm rim around the cavity received between 63% and 84% of the prescribed dose, depending on the size of the applicator. The applied dose was reduced to 38–53% at 5 mm and 18–32% at 10 mm tissue depth. After IORT, the applicator was removed and surgery was completed. After treatment, all patients received standardized FU including 3‑monthly MRI of the brain, according to UKA FU policy for LBRT. All statistical analyses were performed with EZR (Version 3.4.1/The R Foundation for statistical computing, Vienna, Austria) [14] using Kaplan–Meier methods and log-rank tests.

## Results

**Fig. 1:**
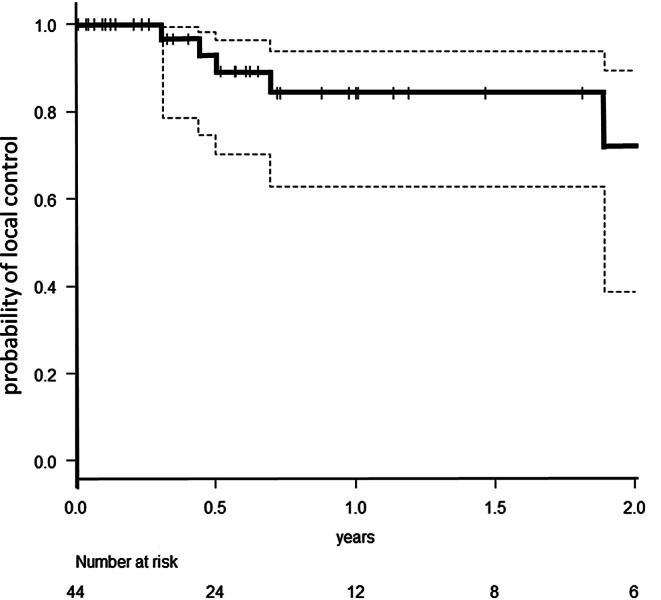
Probability of local control after resection of BM and IORT (the *dotted lines* represent the 95% confidence intervals)

We identified 40 patients (22 female/18 male) with 44 resected BM who had been treated with 50-kV x‑ray IORT. For patients characteristics see Table 1. Median age of these patients at time of treatment was 62.8 years (range 29–83 years). Most patients fitted to recursive partitioning analysis (RPA) [15] class 2 (31 patients; class 1: 6 patients; class 3: 3 patients). Median diameter of the resected metastases was 2.8 cm (range 1.5–5.9 cm). Median applied dose was 20 Gy (range 13.4–20 Gy). Four patients had a history of previous EBRT in the area of resection. All other patients were newly diagnosed with BM prior to resection. Median number of BM at treatment time was one (range 1–6). Maximum number of IORT procedures per patient was two. All other non-resected brain lesions were treated with stereotactic radiotherapy (SRS) with the exception of one patient receiving additional WBI. The predominant histology of the resected metastases was non-small-cell lung cancer (14 metastases), followed by breast cancer (8 metastases) and malignant melanoma (7 metastases). Twenty-four of these patients were simultaneously suffering from additional tumor burden in organs other than brain. After treatment, all patients received standardized follow-up (FU) including 3‑monthly MRI of the brain according to UKA FU policy for LBRT. Mean follow-up was 14.4 months (SD: 18.1 months), with a median MRI follow-up for alive patients of 12.2 months (range 0–58.1 months). At the time of this analysis, 16 of these 40 patients had died. Median overall survival of all treated patients was 26.4 months (range 0.5–73.0 months), with an estimated overall survival at 1 year of 61.6%. The observed local control (LC) rate of the resection cavity was 88.6%, with estimated LC of 84.3% at 1 year (Fig. 1). All recurrences except one were histologically proven (NSCLC, breast cancer, malignant melanoma, ovarian cancer). Observed distant brain control (DC) was 47.5% with estimated DC of 33.5% at 1 year, including 4 patients (10%) who developed leptomeningeal disease (LMD). The estimated LMD rate was 16.2% at 2 years. Only 25% of all patients received WBI in the further course of disease to achieve DC. All other patients could be salvaged via focal treatment. IORT did not increase the perioperative toxicity of brain surgery. Thirty-day mortality of the 44 interventions was 4.5%, not related to IORT. One patient died from sepsis arising of the genitourinary tract and one patient suffered a lethal infarction of the middle cerebral artery 12 days postoperatively. One postoperative bleeding, one postoperative formation of a hygroma, and one wound infection in the area of the craniotomy were observed. One IORT procedure in the posterior fossa had to be terminated prematurely (applied 13.4 Gy of planned 18 Gy) due to detection of an air embolism, which could be treated without consequential damage for the patient. Mean time from surgery to discharge from hospital was 7 days (range 2–27 days). The IORT procedure prolonged OR time by a mean of 25 min (range 15–42 min). Mean operation time including IORT was 166 min (range 97 to 308 min). Mean radiation time was 14:55 min (range 8:13–27:04 min). Twenty-four patients in this series needed systemic treatment due to additional tumor burden in other organs. Median time to start of systemic treatment after surgery was 18 days (range 0 to 130 days) for these patients. Brain necrosis of any grade after IORT was observed and histologically proven in one single, symptomatic case of a lesion in a pre-irradiated area only (observed brain necrosis rate 2.5% /estimated brain necrosis rate of 6.7% at 1 year). None of the non-pre-irradiated patients experienced radiologic signs of brain necrosis.

**Table 1:** Patient and disease characteristics

Patients characteristic	
Number of patients	40
Male/female	18/22
Patients alive	24
Median age (range)	62.8 years (39–83 years)
Patients with previous brain RT	4
Patients with metastases in other organs	24
*RPA*	
Class 1	6
Class 2	31
Class 3	3
*Lesion characteristics*	
Median number of BM at treatment (range)	1 (1–6)
Median size of treated lesion (range)	2.8 cm (1.5–5.9 cm)
Median size of applicator (range)	2.0 cm (1.5–4.0 cm)
Median dose (range)	20 Gy (13.4–20 Gy)
Suspected incomplete resection in MRI	14
*Location of brain metastases*	
Frontal	13
Parietal	8
Occipital	12
Temporal	5
Posterior fossa	6
*Histology of resected metastases*	
NSCLC	14
Breast cancer	8
Melanoma	7
Colorectal carcinoma	6
Renal cell carcinoma	3
Other (Ovarian cancer/parotid cancer/bladder cancer/esophageal cancer)	6

## Discussion

To our knowledge, this is the largest published mono-institutional series of patients treated with IORT after resection of BM. In contrast to previous publications in this field [12, 13] reporting mainly LC/DC and toxicity data, we report a wider scope of the implementation of IORT in the treatment of patients with BM and its impact on multidisciplinary care. In 2013, we started treating patients with IORT after neurosurgical BM resection on an individual case-by-case treatment decision basis after interdisciplinary discussion and decision in the MTB. With increasing evidence for this procedure [12], this treatment is offered routinely to patients planned for resection of BM after MTB recommendation at the UKA as an optional treatment, replacing postoperative external beam LBRT in close to 40% of these patients in the last 3 years of the analyzed period. From the patients’ perspective, this is an attractive option, as it is a “one-stop shop” with no further necessity of adjuvant radiotherapy after discharge from hospital. The observed LC in our series is definitively in the upper range of data reported after brain metastasectomy [3, 16–24]. When we started using IORT for LBRT in 2013, we applied lower doses of 16 Gy according to the Cleveland protocol [12]. After publication of the toxicity data of the INTRAGO phase1/2 study [25], we increased the dose in a stepwise manner and, since 2016, all patients have been irradiated with 20 Gy. Other centers nowadays apply even higher doses [13, 25, 26]. However, with the reported outcome of our patients, we do not currently see a necessity for further dose escalation. Additionally, it has to be taken into account that the relative biological efficacy (RBE) of the applied low-energy x‑rays (LEX) lies between 1.3 and 1.5 [27–29]. This means that 1 Gy applied with 50-kV x‑rays is biologically iso-equivalent to 1.3 to 1.5 Gy applied with photons (RBE 1). This makes 20 Gy of LEX IORT biologically iso-equivalent to 26 to 30 Gy of 6‑MV photon SRS [30].

Within the IORT procedure, the choice of the optimally sized applicator and its correct placement in the resection cavity is of high importance. The applicator must fit into the cavity, providing direct contact of the cavity walls to the surface of the applicator. The treated volume around the resection cavity receiving a therapeutic dose encompasses a seam of tissue of only 5 to 10 mm at maximum [31, 32]. Due to the steep dose gradient of LEX, the choice of an applicator which is smaller than cavity size will result in underdosage of parts of the cavity. Applicator sizes bigger than the cavity space may cause harm to brain tissue by mechanical pressure. For the same reasons, optimal placement of the applicator within the cavity is also a key issue. This is based on the experience of the neurosurgeon only and his knowledge of the operative site, because up to now, there is no option for three-dimensional imaging of the patient with the applicator in the treatment position.

**Fig. 2:**
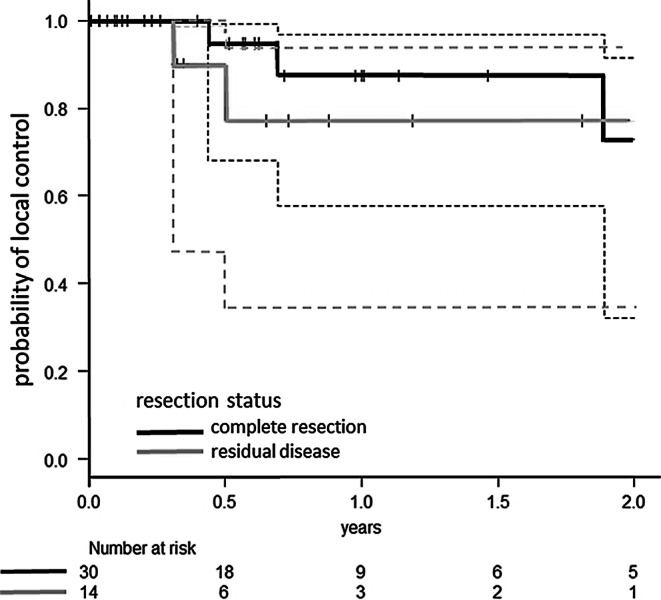
Probability of local control after resection of BM and IORT stratified for complete or incomplete resection of BM in postoperative MRI (the *dotted lines* represent the 95% confidence intervals)

This series includes 14 patients with suspected incomplete resection in post-operative MRI. This raises the question of whether these patients were treated with an insufficient dose intraoperatively. In a similar postoperative EBRT setting, we would have treated the patient with an increased dose [2]. In our analysis, LC of patients with suspected residual disease did not significantly differ from the LC of patients with complete resection on postoperative MRI (87.4% versus 77.1% at 1 year/*p* = 0.473; Fig. 2). However, due to the limited sample size, the validity of this observation might be questioned. However, the applied biological effective dose (BED) of the IORT might be sufficient to control residual disease at the cavity walls in direct contact with the surface of the applicator. Another aspect of this issue is the observation that resection cavities after IORT tend to enhance contrast media more pronouncedly in postoperative MRI compared to cavities after surgery only. This might have led to an overdiagnosis of suspected residual disease in postoperative MRI in patients treated with IORT, blurring the correct stratification. Nonetheless, in the IORT setting, the neurosurgeon should utterly strive for complete resection of the BM.

In this series, more than half of the patients (21 out of 40 patients) experienced DBF after neurosurgical resection of BM and IORT. We assume this is more likely an effect of patient selection than of the specific treatment technique. One-year DBF rates in the literature vary between 20 and 70% [2, 19, 21, 22, 33–36]. Due to FU including 3‑monthly MRI of the brain, all DBF of our patients were diagnosed in an asymptomatic stage. The majority of these cases could be successfully salvaged by SRS. Only 25% of all patients needed WBI due to multiple new BM or LMD in the complete further course of disease to achieve DC. The estimated LMD rate of 16.2% at 2 years in this report matches perfectly with the reported 2‑year LMD rates of patients treated with focal EBRT postoperatively [33, 35, 37, 38]. Hence, there is no evidence that the IORT procedure affects the risk of tumor cell spillage to the cerebrospinal fluid by neurosurgery.

In this series, IORT did not increase the perioperative morbidity and mortality of neurosurgical metastasectomy or the time of hospitalization after surgery [39–41]. The observed radionecrosis (RN) rate in our patients was 2.5%. We observed RN formation only in one single case of a lesion in a pre-irradiated area. None of the non-pre-irradiated patients experienced radiologic signs of RN. In the current literature the rates of RN rage from 5 to 25% for patients treated with HSRT after resection of BM and tend to be a little higher for patients treated with SRS in the same setting [3, 18, 34, 42]. This reasonably low RN rate could possibly be explained by the relatively small volume of surrounding brain tissue receiving 10 Gy (V10), due to the steep dose gradient of LEX. V10 is an established risk factor for RN in SRS. In our series the mean applicator size was 2.0 cm (range 1.5–4.0 cm), corresponding to a nominal mean V10 of 6.12 cm^3^ (range 3.08–35.95 cm^3^). Taking into account RBE, the corresponding mean V10 (RBE) is 12.97 cm^3^ (range 4.6–48.94 cm^3^). In this context, the combination of observed high LC and low RN rates in this series appears to be very favorable. This could only be achieved as a joint interdisciplinary effort of a multiprofessional team.

## Conclusion

IORT with 50-kV x‑rays is a safe and convenient way to apply LBRT after neurosurgical resection of BM. It is associated with low toxicity and excellent LC. The IORT procedure has only a minor impact on total OR time and does not prolong the patients’ recovery time in hospital. For patients with additional tumor burden, IORT LBRT holds the chance for an early start of adjacent systemic therapy. Three-monthly FU with MRI is paramount for LBRT concepts to detect the frequent distant brain failure (DBF) early. In this setting, WBI could be avoided for 75% of the patients in the further course of disease, using SRS as an effective salvage therapy for DBF.
